# Boat-shaped houses of the indigenous Li people on Hainan Island, China: plant resources and ecological adaptations

**DOI:** 10.1186/s13002-025-00818-9

**Published:** 2025-10-14

**Authors:** Guang-Hui Ma, Ming-Xun Ren, Ding-Hai Yang, Xiao-Dong Mu

**Affiliations:** 1 International Joint Center for Terrestrial Biodiversity Around South China Sea of Hainan Province, School of Ecology, Hainan University, Haikou, 570228 China; 2 School of Tropical Agriculture and Forestry, Hainan University, Haikou, 570228 China; 3 Hainan Research Academy of Environmental Sciences, Haikou, 571126 China

**Keywords:** Boat-shaped house, Adaptation, Tropical rainforest plants, Indigenous Li people, Traditional knowledge, Vernacular architecture

## Abstract

**Background:**

The traditional boat-shaped houses of the Li people on Hainan Island, China, reflect centuries of ecological adaptation to the tropical rainforest. These vernacular dwellings are now threatened by rural depopulation and rapid modernization. We explore the ecological function, material use, and cultural value of the boat-shaped houses of the Li people and support their nomination as a site of UNESCO World Natural and Cultural Heritage.

**Methods:**

We combined ethnobotanical surveys, environmental measurements, and literature analysis to evaluate plant-based construction, house–environment interactions, and traditional knowledge.

**Results:**

The study identified four types of traditional boat-shaped houses of the Li people on Hainan Island, constructed using 26 plant species across 13 families. Environmental monitoring showed that in natural conditions, boat-shaped houses had lower indoor air temperatures (by 1.3 °C in Chubao Village), reduced relatively humidity (by 7.3% in Baicha Village), and significantly lower wet bulb globe temperatures (by 9.6 °C in Baicha Village), compared to modern brick houses. Boat-shaped houses thus provided a more thermally comfortable environment than modern brick houses, particularly during the dry season. The results emphasize the green, low-carbon construction cycle of boat-shaped houses and highlight the urgent need to conserve this ecologically sustainable traditional knowledge system.

**Conclusions:**

Li boat-shaped houses demonstrate a low-carbon, climate-adaptive building system rooted in indigenous knowledge. Their preservation offers critical insights for sustainable design and biocultural conservation in tropical regions.

**Supplementary Information:**

The online version contains supplementary material available at 10.1186/s13002-025-00818-9.

## Background

Traditional knowledge (TK) plays a vital role in the conservation of contemporary biodiversity and human well-being [1, 2]. For generations, indigenous and local communities have utilized time-tested practices to maintain ecological balance, ensuring the sustainable use of natural resources. This knowledge supports biodiversity conservation, soil fertility, and water management, which are essential for resilient ecosystems [3, 4]. By integrating indigenous wisdom with modern science, societies can develop holistic strategies to address global challenges such as climate change, biodiversity loss, and food insecurity. Preserving and valuing traditional knowledge is thus essential for a sustainable and equitable future [5]. Utilization of plant resources among different ethnic groups or indigenous communities in southwest China becomes a research hot spot beyond ethnobotany and social sciences [6–14].

Among various forms of TK, vernacular architecture is particularly important as it reflects the ecological adaptation and livelihood strategies of local communities [15]. There are many kinds of tropical vernacular architectures still popular in traditional villages in the world, such as longhouses and floating houses in the Amazon area, Dai bamboo stilt houses in China, and tongkonan in Indonesia. Chandel reviewed the vernacular architectures in Australia, New Zealand, South Africa, France, New Mexico, Colombia, Spain, and India, assessing their suitability in different climatic zones, and identifying their thermal comfort, energy-efficient features, passive solar features, current design and construction techniques. It is possible and promising to utilize vernacular materials and architectural features for improving thermal comfort in modern buildings worldwide. The stilt-style architecture represents a distinctive example of such adaptation in tropical and subtropical regions, particularly in Southeast Asia [16–19]. But there is almost no scientific research about stilt-style architecture in Hainan, the only province in China that is entirely located in the tropical zone, excepting some descriptive articles in Chinese social and artistic aspects.

Hainan Island, located in southern China and separated from the mainland by the Qiongzhou Strait, is home to the indigenous Li people, the first known inhabitants of the island, who speak Li language, with five dialects (Ha, Qi, Run, Sai, and Meifu) in different zones [20]. For more than three thousand years, the Li people have lived in close proximity to tropical rainforest, developing a unique architectural form, the boat-shaped house, so named its roof is shaped like an upside-down wooden boat. According to the traditional legend of the Li people, the origin of the boat-shaped house is closely tied to the migration story of their ancestral figure, Princess Danya. It is said that she arrived on Hainan Island in a wooden boat. With no available shelter upon arrival, she overturned the boat and used it as a makeshift dwelling. This improvised structure later inspired the development of the Li people’s distinctive vernacular architecture and marked the beginning of their settlement on the island. Their architecture is adapted to the island’s mountainous terrain, hot and rainy climate, frequent typhoons, and rich biodiversity. The boat-shaped house serves both as a dwelling and as a cultural symbol, deeply embedded in the social and ecological systems of the Li people [21]. Through generations of coexistence with the rainforest ecosystem, the Li people have developed diverse subsistence strategies, such as collecting wild edible and medicinal plants, fishing, hunting, and swidden agriculture. They also practice weaving and pottery making. In all these practices, plant resources have played an essential role, particularly in the construction of boat-shaped houses, which use a wide variety of locally sourced materials such as wood, bamboo, rattan, and grass. Despite their ecological and cultural significance, detailed documentation of specific plant species used and construction knowledge remains limited. Most elderly Li people who are the key knowledge holders do not speak Mandarin and much of the traditional knowledge (TK) they possess is at risk of disappearing amid urbanization and tourism development. This study aims to investigate the thermal environmental performance, construction logic, and ethnobotanical knowledge embedded in traditional boat-shaped houses of the Li people on Hainan Island. Drawing on cross-cultural comparisons with vernacular architecture in other tropical and subtropical regions, the study seeks to:

1. Analyze how locally available plant species are selected and used in response to climatic and ecological constraints;
2. Compare the thermal comfort performance of traditional and modern house types in different environmental and topographical settings;
3. Explore how traditional architectural knowledge contributes to sustainability and passive climate adaptation strategies.

Through this multi-scalar and interdisciplinary approach, we aim to highlight the broader implications of indigenous architecture for climate-responsive design in humid tropical regions worldwide.

## Methods

### Study area

**Fig. 1:**
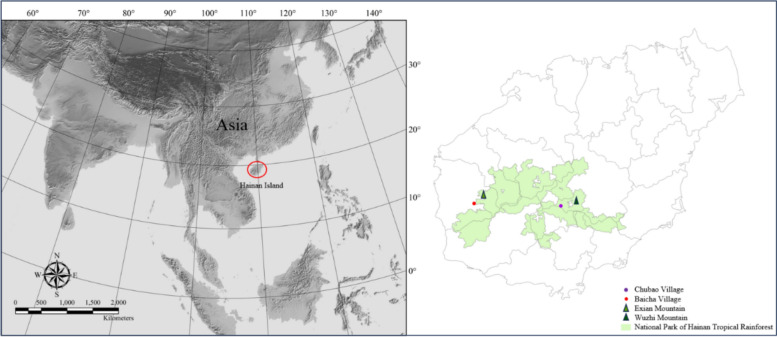
Geographic locations of villages with traditional Li boat-shaped houses: relative positions of two Li villages and the Hainan Tropical Rainforest National Park on Hainan Island in Asia

This study was carried out in Chubao Village (18°50′N, 109°35′E) and Baicha Village (18°55′N, 108°55′E), the only two remaining settlements in Hainan where the Li people still inhabit traditional boat-shaped houses. Both villages have a history of more than 100 years. Chubao Village is located in the south-central mountainous region of Hainan Island, within Maoyang Township near Wuzhi Mountain. It is inhabited by Qi dialect speakers, with 58 households and 320 residents. The village contains 36 well-preserved boat-shaped houses with wooden walls, surrounded by valley lowland rainforest. The boat-shaped houses in Chubao Village are built along the mountain slope, with some located on the hillside and others at the foot of the hill, forming a distribution across different elevation gradients. The house orientations are generally consistent. The village is located at about 450 m altitude, and the regional climate is tropical rainforest. The area receives approximately 1800 mm of annual precipitation, 1,829 h of sunshine, and maintains a mean annual mean temperature of 24.1 °C and relative humidity of 80%. Baicha Village is located in Jiangbian Township, Dongfang City, near Exian Mountain. It is home to 83 households and 384 people who speak the Meifu dialect. There are 81 remaining boat-shaped houses in Baicha, characterized by mud walls reinforced with internal wooden frames. There are two types of orientation of houses: east–west and north–south. The area lies in an intermontane basin at 140 m altitude with a tropical monsoon climate, with an average annual temperature of approximately 24.5 °C, relative humidity around 86%, annual precipitation averaging 1700 mm, mostly concentrated in the wet season from May to October, and 2000 h of sunshine annually [22–24]. These geographic and climatic conditions make the two villages’ favorable environments for studying traditional settlement and construction practices (Fig. 1, Table 1).

**Table 1:** Basic information of two villages with traditional li boat-shaped houses

Attribute	Chubao village	Baicha village
Language	Li	Li
Li Dialect	Qi	Meifu
No. of Houses	36	81
Altitude	450 m	140 m
Geographic Coordinates	18.9876°N, 109.5203°E	18.9211°N, 109.6108°E
Ecological Zone	River valley rainforest	Basin between mountains
Climate Type	Tropical forest	Tropical monsoon
Settlement History	Relocated over 50 years ago	Inhabited for over 100 years
Religion	Animism	Animism
Population	320 people	384 people
No. of Households	58	83
Study Participants	110 (70 female, 40 males; age 25–65;13 informants)	246 (152 females, 94 males; age 30–73; 29 informants)

## Data collection

### Ethnobotanical investigation

Fieldwork was carried out over four time periods: March 2022, April 2023, January and July 2024. Ethnobotanical surveys were conducted in both villages. The main research methods included free listing, semi-structured interviews, participatory observation, and interviews with key informants. A total of 42 key informants were selected from 356 participants using snowball sampling. If a participant knew how to build the boat-shaped house, then he or she was selected to go on to the next steps as a key informant. Informants were first asked to freely list plants used in construction of boat-shaped houses, followed by semi-structured interviews. The interviews were conducted in Mandarin and translated into the Li language with the help of local guides. The questions asked in semi-structured interview were as follows:

1. Plant species identification

What specific plants are traditionally used to construct boat-shaped houses?

(Probe: local names, parts used—e.g., leaves, stems, wood types).

(2) Sourcing locations

Where are these plants typically collected?

(Probe: forest areas, cultivated sources, distance from village).

(3) Seasonal Timing

When is the optimal time to harvest these plants?

(Probe: Seasonal restrictions, lunar calendar connections).

(4) Material selection criteria

Which plant materials are considered superior for construction and why?

(Probe: durability, flexibility, water resistance).

(5) Construction process

Could you describe the step-by-step process of building a boat-shaped house?

(Probe: Tools used, community roles, time duration).

(6) Cultural Values

Why do the Li people prefer boat-shaped houses over other structures?

(Probe: spiritual meaning, historical identity, practical benefits).

(7) Preservation challenges.

What are the main threats to maintaining this tradition today?

(Probe: Material scarcity, lack of builders, disinterest of youth).

These seven questions encompass the various spatial and temporal elements and related traditional knowledge required for constructing Li people’s boat-shaped houses, including local plant species names, distribution areas, collection times, harvesting methods, plant parts used, usage frequency (how often a species was mentioned), abundance (plants used easy to find or not in the wild), specific construction steps, and social impacts. In this study, frequency and abundance data were obtained through participatory methods, including semi-structured interviews and group discussions with local informants. Frequency was calculated as the percentage of informants who mentioned a particular species relative to the total number of informants:

Frequency (%) = (Number of informants who cited the species/Total number of informants) × 100. Abundance, in the context of local perception, was assessed based on informants’ subjective evaluation of the species’ availability or visibility in the surrounding environment. A 5-point Likert scale (1 = very rare, 5 = very abundant) was used to quantify perceived abundance, and the mean score across all informants was taken as the abundance value [25].

### Ethical considerations

There is no research ethics committee or other review procedure for research conducted at Hainan University. However, the Hainan Forestry Bureau approved the study and issued a research permit (QF(NO.2024119). Construction processes were documented with photographs and videos upon permission given by participants. We obtained informed consent verbally from all participants. After obtaining permission, photographs and videos were taken that show the main steps in the building boat-shaped houses and other related details.

### Plant identification

The researchers, accompanied by the villagers, collected plant species that were used in construction of boat-shaped house. Identification was aided by photographs from the Plant Photo Bank of China (http://ppbc.iplant.cn/). The nomenclature followed the *Flora of China*, *Hainan Plant Species Diversity Inventory*, and the Illustrated Handbook of Plants in tropical Rainforest Area of China: Plants of Hainan [26–28]. Identifications were confirmed by Ming-Xun Ren, and voucher samples were deposited at the Herbarium of the School of Ecology in Hainan University.

### Types of boat-shaped houses identification

**Fig. 2:**
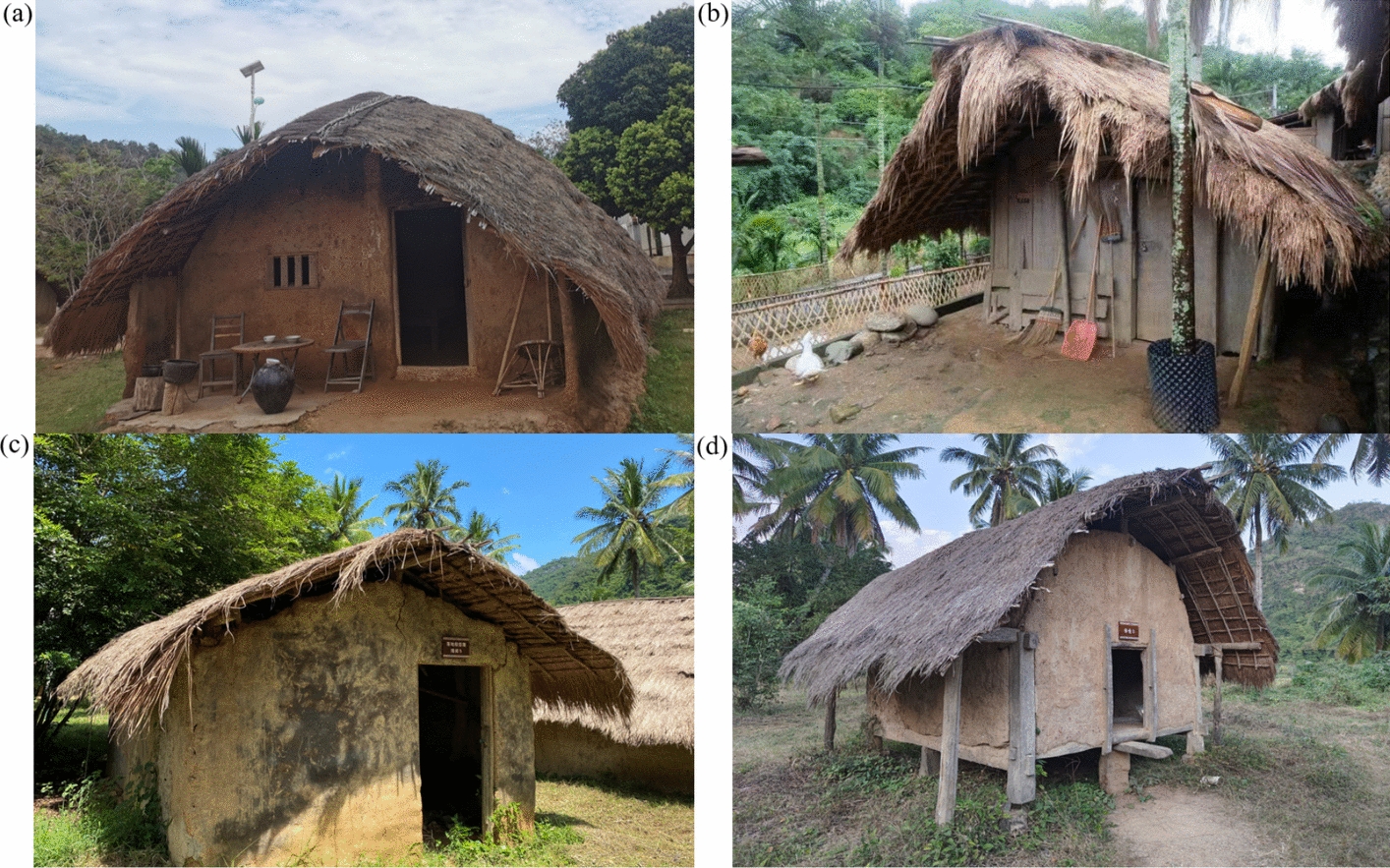
Main structural types of Li boat-shaped houses in two study villages. **a** Residential boat-shaped house with timber frames and mudwalls; **b** Residential boat-shaped house with wooden walls; **c** Long boudoir; and **d** Granary

Four traditional types of boat-shaped houses were identified at the two study sites. Boat-shaped houses with timber frames and mudwalls only exist in Baicha village, where they served as family residential houses. Residential boat-shaped houses with wooden walls only remain in Chubao village, where they also serve as family residences. Long boudoirs are a third type of boat-shaped house, which are specifically designated as places where unmarried young girls reside and rest. Houses of this type are found in both villages. Their layouts and interiors are simple, with no facilities for cooking or fire-making activities. Their structure is similar to that of residential boat-shaped houses, but they are smaller in size and each typically accommodates no more than two or three young girls. Granaries are a fourth type of boat-shaped house, primarily used for storing grain and cereals. Granaries are typically built in an elevated, stilted design and are often situated at a certain distance from residential areas to facilitate fire prevention and management of grain production and harvesting. Granaries are small in size, allowing only a single adult to enter in a crouched position. Both villages contain several granaries of similar construction (Fig. 2). Additionally, modern brick houses in both villages typically feature concrete foundations, cement brick walls approximately 25–30 cm thick, a reinforced concrete roof, and corrugated metal sheets, installed as an additional layer above the reinforced concrete roof. These buildings generally have larger windows and doors than traditional boat-shaped houses. Most brick houses are oriented similarly to traditional dwellings, but without specific consideration of wind direction or solar exposure during construction.

### Collection of traditional architectural building practices

**Fig. 3:**
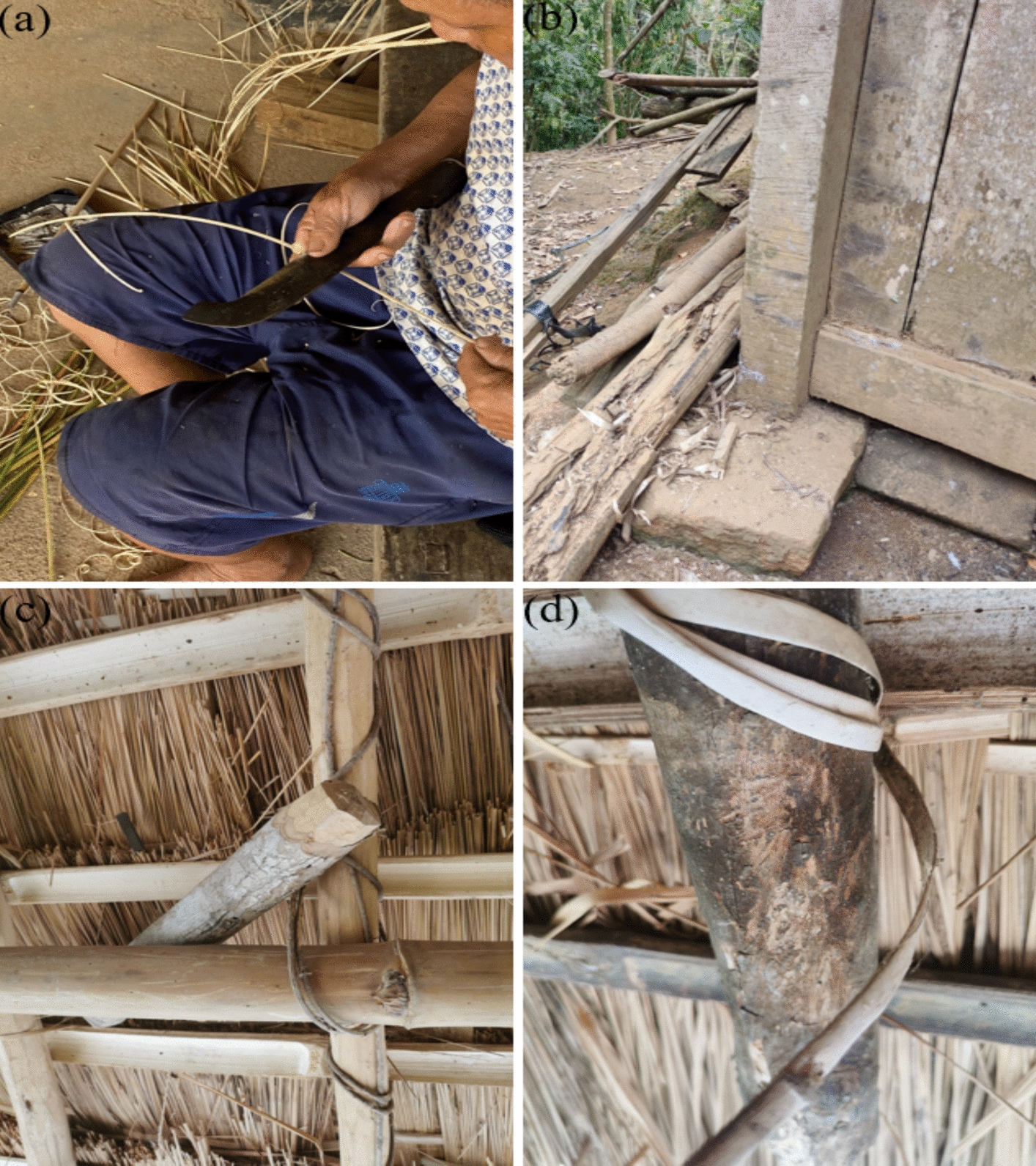
Practices of building boat-shaped houses in traditional Li villages. **a** handcrafting building materials with local wild plants; **b** using stems of trees as beams and columns; **c** using lianas to consolidate the heavier parts of the boat-shaped house; and **d** binding the framework with strips of bamboos

By conducting semi-structured interviews and participatory investigations, we sought to capture the detailed traditional knowledge embedded in every stage of the construction process of boat-shaped houses. The construction process includes several key stages: site selection, material preparation, foundation laying, framework assembly, roof thatching, and door installation, each involving specific forms of traditional knowledge and skills, such as handcrafting building materials with local wild plants, using stems of trees as beams and columns, using lianas to consolidate the heavier parts of the boat-shaped house, and binding the framework with strips of bamboos (Fig. 3).

### Indoor temperature and humidity measurement in boat-shaped houses

Indoor environmental performance was assessed by measuring air temperature, relative humidity, and wet bulb globe temperature (WBGT)—key variables for thermal comfort and evaluation of health risks [29–34]. Due to logistical constraints and the lack of electricity in some houses, we only collected environmental data during daytime hours (9:00–18:00). The absence of night-time data is a recognized limitation of this study.

Measurements were continuously recorded from 9:00 am to 6:00 pm for two days in both the dry (January) and rainy (July) seasons of 2024. Data were collected including indoors and outdoors, and across building types and orientations in Baicha village, and across building types and elevations in Chubao village.

According to the Evaluation Standard for Indoor Thermal and Humid Environment of Civil Buildings in China (GB/T 50785-2012), the types of houses and specific building numbers to be measured, as well as the measurement methods, were determined after extensive surveys and experimental trials [35]. Formal measurements of key thermal environmental parameters, including air temperature, relative humidity, and WBGT, were conducted in January and July of 2024. Due to the limited availability of traditional house types and suitable measurement conditions, a total of only 21 buildings were selected for field monitoring in the two villages.

Each building is comprised of a single room. For each building, the geometric center of the floor was designated as the origin (Point 0). Based on a plum blossom-inspired sampling method, five additional measurement points were symmetrically arranged around a central point on the same horizontal plane. The point aligned with the main entrance was designated as Point 1, and the remaining four points were numbered sequentially as Points 2 through 5 in a counterclockwise direction. At each of the five selected points within a room, measurements were taken at three vertical heights: 0.3 m, 0.9 m, and 1.7 m, resulting in 27 data points per building. Ultimately, two datasets—one for the dry season and one for the rainy season—were obtained from the 21 buildings. To account for variations across different vertical measurement heights, data from each time point were converted into weighted values based on the relative differences among the three heights (Table 2).

**Table 2:** Number of measured house samples in Chubao and Baicha Villages

House samples	Chubao (n)	House ID(s)	Baicha (n)	House ID(s)	Total (n)
Traditional Houses					
Granaries	2	CBG1, CBG2	3	BCG1,BCG2, BCG3	5
Long Boudoirs	2	CBLB1, CBLB2	3	BCLB1,BCLB2, BCLB3	5
Residential Boat-shaped houses	3	CBRB1, CBRB2, CBRB3	4	BCRB1,BCRB2,BCRB3,BCRB4	7
Modern Houses					
Brick houses	2	CBB1,CBB2	2	BCB1,BCB2	4
Total	9		12		21

CBG—granaries in Chubao Village, BCG—granaries in Baicha Village, CBLB**—**long boudoirs in Chubao Village, BCLB—long boudoirs in Baicha Village, CBRB**—**residential boat-shaped houses in Chubao Village, BCRB**—**residential boat-shaped houses in Baicha Village, CBB—brick houses in Chubao Village, BCB—brick houses in Baicha Village; residential boat-shaped houses are different between the two villages** (**wooden walls in Chubao, timber and mud walls in Baicha)

To derive a representative indoor thermal condition at each time point, a weighted average temperature was calculated across the three vertical measurement heights (0.3 m, 0.9 m, and 1.7 m). The formula is as follows:

$${\text{T}}_{{{\text{weighted}}}} = \omega_{{{\text{i1}}}} {\text{T}}_{{0.{3}}} + \omega_{{{\text{i2}}}} {\text{T}}_{{0.{9}}} + \omega_{{{\text{i3}}}} {\text{T}}_{{{1}.{7}}}$$

where T_0.3_,T_0.9_, and T_1.7_ represent the average temperatures at 0.3 m, 0.9 m, and 1.7 m heights, respectively. The weights ${\omega }_{1}$, ${\omega }_{2}$, and ${\omega }_{3}$ were determined based on the inverse of the variance observed across six spatial measurement points at each height, normalized such that

$$\omega_{1} + \omega_{2} + \omega_{3} = 1$$

Subsequently, the mean of the weighted indoor temperatures across all time points was calculated for each house type to represent its daytime indoor temperature, which was then used as the variable in independent samples t tests.

$$\omega_{h} = \frac{{\frac{1}{{\sigma_{h}^{2} }}}}{{\mathop \sum \nolimits_{j = 1}^{3} \frac{1}{{\sigma_{j}^{2} }}}}\quad \left( {h = 1,2,3} \right)$$

$$\overline{T}_{{{\text{type}}}} = \frac{1}{n}\mathop \sum \limits_{i = 1}^{n} \left( {\mathop \sum \limits_{h = 1}^{3} \omega_{h} \cdot \frac{1}{6}\mathop \sum \limits_{k = 1}^{6} T_{ihk} } \right)$$

The average weighted indoor temperature for each house type during the daytime, denoted as ${\overline{T}}_{\text{type}}$, was calculated using all temperature measurements from the three height levels. At each time point i, the temperature from six sensors at each height level h was first averaged to obtain a representative value for that height. These height-specific means were then combined using their corresponding weights ${\omega }_{h}$​, resulting in a weighted temperature for that time point. The final daytime average was obtained by averaging the weighted values across all n = 9 time points.

The weight ${\omega }_{h}$ for each height level was calculated as the inverse of the variance of the temperature measurements at that height, normalized by the sum of the inverse variances of all three heights. This approach gives more influence to measurements from height levels with lower variability, reflecting higher measurement reliability.

We used independent-sample t tests to compare the mean thermal indices (air temperature, relative humidity, globe temperature, and WBGT) between traditional and modern house types, separately for each village and season. Statistical significance was assessed at the 0.05 level, and standard errors were calculated to evaluate the consistency of measurements across replicate houses of the same type.

## Results and analysis

### Building processes and plant species used in construction of boat-shaped houses

The construction of traditional boat-shaped houses follows a series of coordinated steps that integrate practical craftsmanship with deep-rooted ethnobotanical knowledge. The process typically begins with site selection and land clearing, after which a simple foundation is laid using local materials.

The primary frame and structure are erected using durable hardwoods for columns and beams. Species commonly used include *Liquidambar formosana* (Altingiaceae), *Erythrophleum fordii* (Fabaceae), and *Homalium ceylanicum* (Salicaceae), which are favored for their strength and resistance to decay. Other species such as *Litchi chinensis* (Sapindaceae), *Melia azedarach* (Meliaceae), *Madhuca hainanensis* (Sapotaceae), and *Dalbergia hainanensis* (Fabaceae) are also selected depending on local availability and builder preference.

Framing members, especially for the sidewalls and roof skeleton, are typically constructed using various bamboo species for their flexibility, light weight, and rapid growth. Commonly used species include *Bambusa bambos* (Poaceae), *Bambusa textilis* (Poaceae), *Lingnania intermedia* (Poaceae), and *Bambusa xiashanensis*. These are split, shaped, and tied into curved profiles that resemble an inverted boat hull.

For binding and joining structural elements, natural fiber materials are preferred over nails or modern fasteners. Rattan species such as *Calamus tetradactylus* (Arecaceae), *Calamus simplicifolius* (Arecaceae), and flexible vines like *Urceola huaitingii* (Apocynaceae), as well as *Dinochloa multiramora* (Poaceae), are used extensively due to their tensile strength and pliability.

Once the frame is completed, roof covering is applied. The most frequently used material is *Imperata cylindrica* (Poaceae), a hardy grass with excellent thatching properties. In some cases, leaves of *Livistona chinensis* (Arecaceae) are also used as supplementary roofing material.

The final step involves internal layout, flooring, and weatherproofing, often with adjustments based on seasonality and household size. Throughout the construction process, plant selection is not only based on availability but also on functional properties such as water resistance, weight, and ease of renewal—reflecting generations of empirical knowledge embedded in Li architectural traditions.

After these steps, the beautiful boat-shaped house is completed, and the whole community of local Li people will celebrate it in a big housewarming party for several days. They drink with the owner of the new boat-shaped house, sing traditional folk songs, and dance together. According to the traditional custom, special wizards are invited to hold religious rites to exorcize bad ghosts and to pray for happiness for those living in the new boat-shaped house. This kind of custom has lasted for thousands of years. Some folk housewarming songs were created and sung by local communities using their language long ago. One sentence in the lyrics of a song: “building a boat-house on a hillside. You can’t get enough of looking at it because of deep love. You drink spring water when you’re thirsty, and you eat betel nuts when your mouth is sour.”

**Fig. 4:**
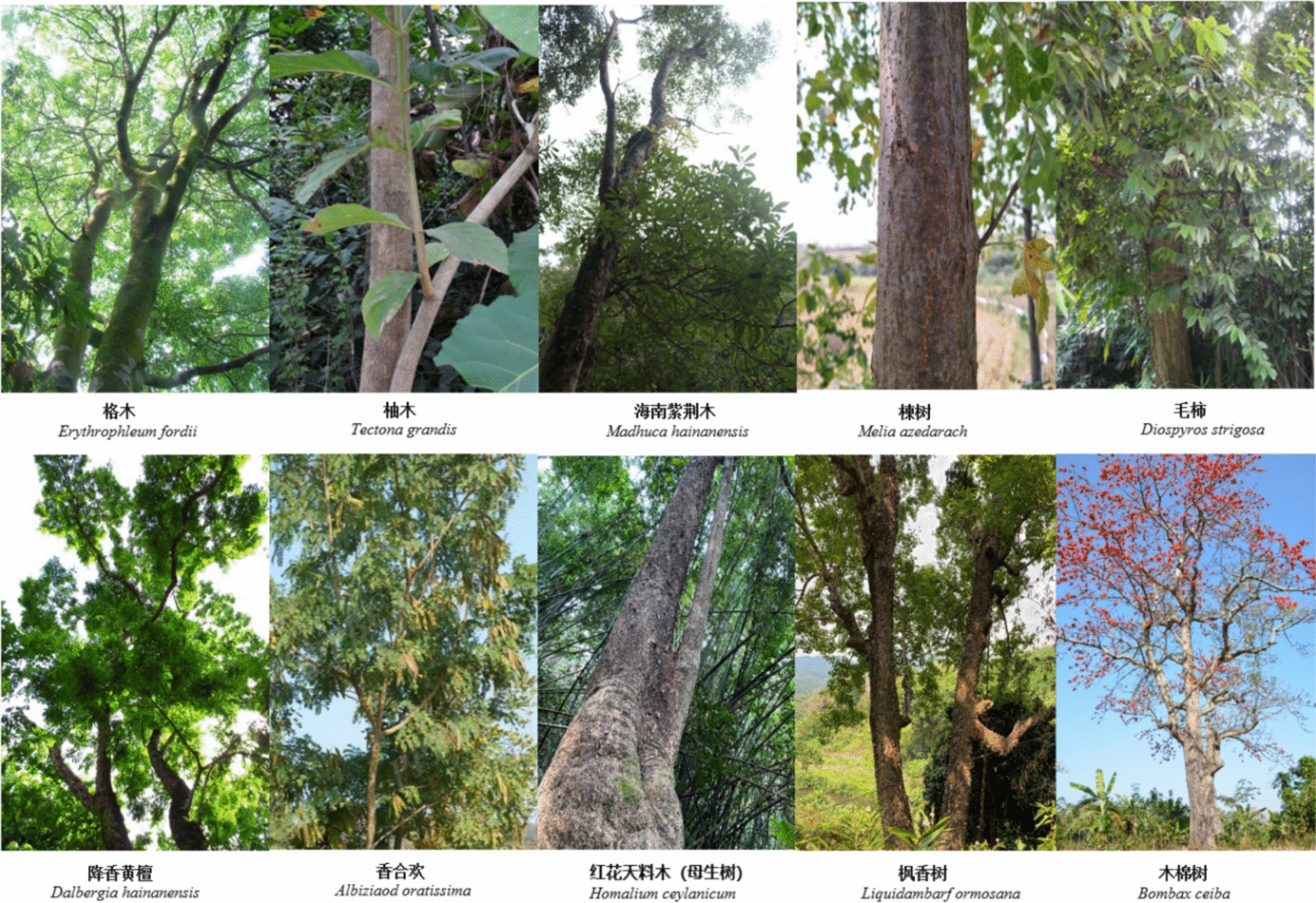
Ten main tree species used in the construction of boat-shaped houses in Li villages

**Fig. 5:**
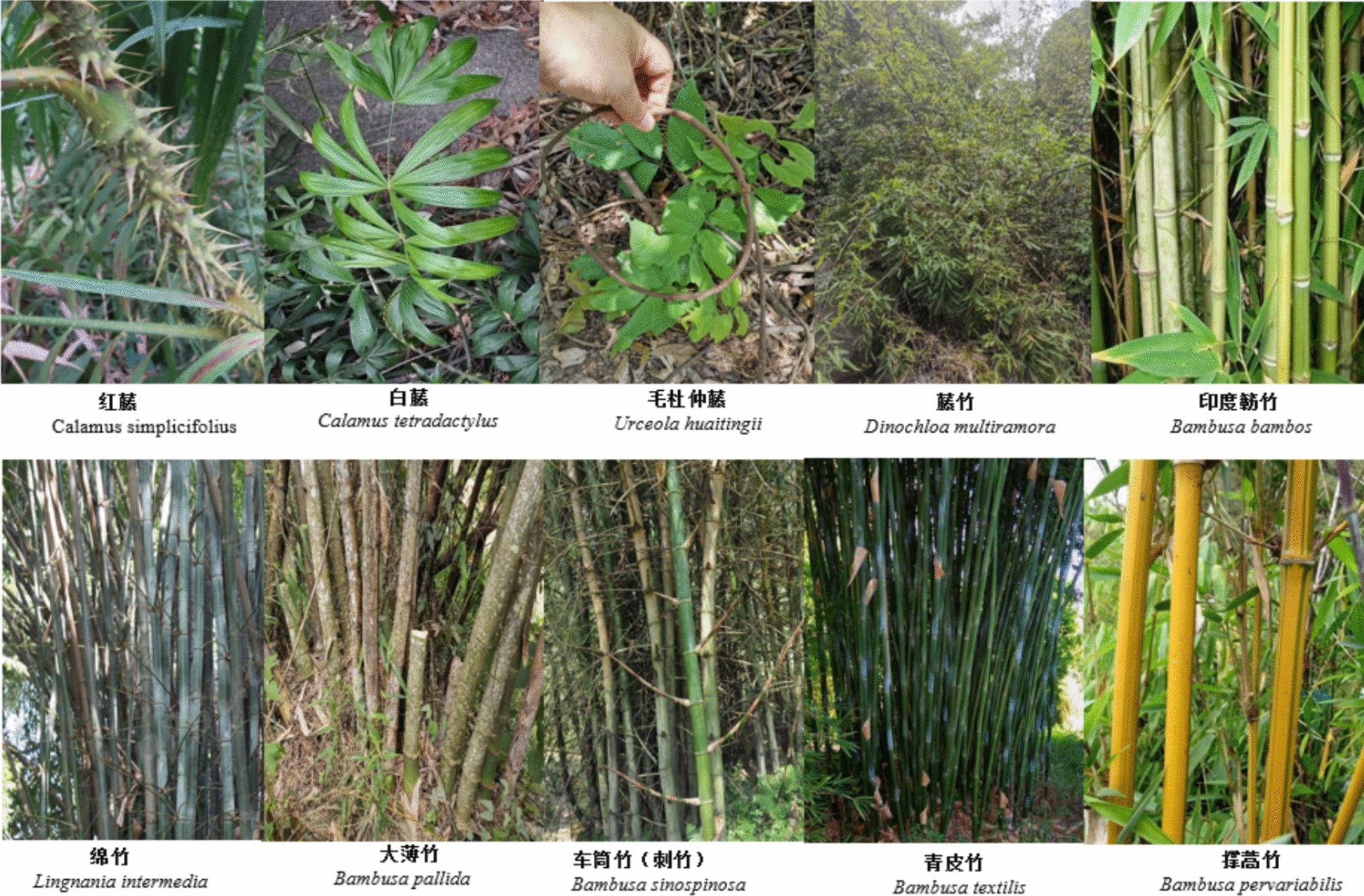
Main species of lianas (including rattans) and bamboos used in construction of boat-shaped houses in Li villages

A total of 26 plant species were documented as used in construction of the houses, including 13 tree species, 9 bamboo species, 3 liana species, and 1 herbaceous species, belonging to 13 botanical families. Each species or plant part was selected for a specific architectural function: Leaves were used mainly for the thatching of the roof, stems for beams, columns and frames, strips of bamboos and rattans for the binding or affixing of components (Table 3; Figs. 4, 5).

**Table 3:** Plant species used in construction of traditional boat-shaped houses by the Li people in Hainan

Utilization	Scientific name	Family	Vernacular	Frequency (%)	Abundance	Plant Part	Voucher code
Roof covering	*Imperata cylindrica* (L.) Raeusch	Poaceae	Mao	100.0	3	Leaf	GHMa-107
	*Livistona chinensis* (Jacq.) R. Br. ex Mart	Arecaceae	Shan	19.0	4	Leaf	GHMa-083
Binding	*Urceola huaitingii* (Tsiang & P.T. Li) D.J. Middleton	Apocynaceae	Dai Yao Fan	100.0	4	Stem	GHMa-142
	*Calamus tetradactylus* Hance	Arecaceae	Bai Fan	100.0	3	Strip	GHMa-096
	*Dinochloa multiramora* S. Dransf	Poaceae	Teng Lao	100.0	4	Strip	GHMa-153
	*Calamus simplicifolius* Wei	Arecaceae	Hong Fan	100.0	4	Stem	GHMa-088
Columns and beams	*Litchi chinensis* Sonn	Sapindaceae	Li Zi	40.5	2	Stem	GHMa-112
	*Erythrophleum fordii* Oliv	Fabaceae	Ji Lai	59.5	3	Stem	GHMa-067
	*Tectona grandis* L. f	Lamiaceae	Shi Yan	19.0	1	Stem	GHMa-175
	*Albizia odoratissima* (L. f.) Benth	Fabaceae	Hei Ge	40.5	1	Stem	GHMa-091
	*Liquidambar formosana* Hance	Altingiaceae	Cai Wao	100.0	4	Stem	GHMa-134
	*Homalium ceylanicum* (Gardner & Champ.) Benth	Salicaceae	Mai Tian Liao	81.0	1	Stem	GHMa-119
	*Madhuca hainanensis* Chun & F.C. How	Sapotaceae	Ke Zai	59.5	1	Stem	GHMa-162
	*Cocos nucifera* L	Arecaceae	Zhe Zun	19.0	5	Stem	GHMa-095
	*Melia azedarach* L	Meliaceae	Zhi Weng	59.5	3	Stem	GHMa-186
	*Bombax ceiba* L	Malvaceae	Ken Hao	19.0	1	Stem	GHMa-078
	*Diospyros strigosa* Hemsl	Ebenaceae	Ze Bao	40.5	1	Stem	GHMa-145
	*Dalbergia hainanensis* Merr. & Chun	Fabaceae	Hua Li Mu	40.5	2	Stem	GHMa-099
Framing	*Bambusa sinospinosa* McClure	Poaceae	Nabo	40.5	1	Stem	GHMa-122
	*Bambusa textilis* McClure	Poaceae	Dam	59.5	3	Stem	GHMa-168
	*Bambusa pervariabilis* McClure	Poaceae	Lao Yanu	40.5	2	Stem	GHMa-108
	*Dendrocalamus membranaceus* Munro	Poaceae	–	40.5	2	Stem	GHMa-131
	*Bambusa pallida* Munro	Poaceae	–	40.5	2	Stem	GHMa-084
	*Lingnania intermedia* (H.L. Li) McClure	Poaceae	Nabo	59.5	3	Stem	GHMa-160
	*Bambusa bambos* (L.) Voss	Poaceae	Bu Lao	100.0	5	Stem	GHMa-174
	*Bambusa xiashanensis* W.T. Lin	Poaceae	–	81.0	4	Stem	GHMa-115

Strip means the slat cut from the stem of that plant, and/-these bamboo species are used by local people, but do not have vernacular names. Numbers 1–5 are the scores given by 42 key informants based on their subjective perception of the species’ abundance and ease of acquisition in the tropical rainforest

Several species served as key materials in multiple functional domains. For example, *Imperata cylindrica* (Poaceae) and *Livistona chinensis* (Arecaceae) were the plants whose leaves were most commonly used for thatch to cover the roof. Binding was achieved using stems and strips of *Urceola huaitingii* (Apocynaceae), *Calamus tetradactylus* (Arecaceae), *Dinochloa multiramora* (Poaceae), and *Calamus simplicifolius* (Arecaceae). Hardwoods such as *Erythrophleum fordii* (Fabaceae), *Dalbergia hainanensis* (Fabaceae), and *Litchi chinensis* (Sapindaceae) were frequently used for structural support (e.g., columns and beams).

This study identified both unique and shared patterns of plant use among indigenous communities, consistent with prior ethnobotanical research. For example, *Calamus* species are commonly used for construction and handicrafts in tropical Asia, as confirmed in recent studies [36–38]. Similarly, the use of *Imperata cylindrica* leaves for roofing aligns with traditional practices documented in West Africa rural communities [39]. However, this study highlights the novel utilization of *Madhuca hainanensis* as a structural timber, a use less commonly reported in the literature, suggesting localized adaptation to available flora. Such findings underscore ecological and cultural drivers behind regional variation in ethnobotanical knowledge [40].

Ethnobotanical investigation shows that the construction of boat-shaped houses by the Li people relies heavily on specific plant materials that are primarily gathered from nearby mountainous areas, particularly in forested zones surrounding the villages. These resources include hardwoods for beams and columns, flexible vines and bamboo for structural frames and binding, and leaves for thatch roofing—each selected for its functional properties such as strength, flexibility, resistance to termites and rotting, and water resistance. Harvesting typically follows seasonal patterns aligned with the lunar calendar, with most materials collected during the dry season to ensure optimal durability and ease of processing due to dryness. Local knowledge plays a key role in determining the appropriate harvesting time and in identifying superior species based on criteria such as longevity, resistance to pests, and ease of manipulation. The Li people’s preference for boat-shaped houses is rooted not only in their climatic adaptability—providing ventilation, insulation, and structural efficiency—but also in their cultural and spiritual significance, as these structures symbolize ancestral heritage and identity. However, the continuity of this tradition faces mounting challenges, including the depletion of key plant species due to environmental change, declining transmission of craftsmanship, and reduced interest among younger generations, many of whom now favor modern brick structures over traditional forms.

According to the National List of Protected Wild Plants and the IUCN Red List, five of the tree species recorded: *Erythrophleum fordii*, *Dalbergia hainanensis*, *Tectona grandis*, *Litchi chinensis* (wild), and *Madhuca hainanensis*, are listed as nationally protected in China (Table 4). *Homalium ceylanicum* is listed as a provincially protected species. In particular, *Madhuca hainanensis*, *Diospyros strigosa*, and *Dalbergia hainanensis* are endemic to Hainan Island. Due to increasing habitat degradation and anthropogenic pressures, many of these species have become rare in tropical rainforest ecosystems [41–43].

**Table 4:** Five nationally protected plant species used in construction of boat-shaped houses

Scientific name	Protection level (China)	IUCN Red list status	Endemic status
*Litchi chinensis*	National Level II	Vulnerable (VU)	Asia Endemic
*Erythrophleum fordii*	National Level I	Endangered (EN)	Asia Endemic
*Madhuca hainanensis*	National Level II	Endangered (EN)	Hainan Island Endemic
*Diospyros strigosa*	National Level I	Critically Endangered (CR)	Hainan Island Endemic
*Dalbergia hainanensis*	National Level II	Critically Endangered (CR)	Hainan Island Endemic

### Collection of performance data for plant materials in boat-shaped houses

The mechanical and ecological properties of plant-based materials used in the construction of Li people’s boat-shaped houses were evaluated on the basis of literature previously published and field data [44–47]. Different construction functions, including roof, structural support, framing, and binding, require different material characteristics (Table 5, Table 6).

**Table 5:** Comparison of mechanical properties of materials (leaves) used for roof covering of boat-shaped houses

Property	*Imperata cylindrica*	*Livistona chinensis*
Air-dry density (g/cm^3^)	0.6–0.8	0.8–1.0
Volumetric shrinkage (%)	6–9	3–6
Tangential shrinkage (%)	4–7	3–5
Radial shrinkage (%)	3–5	2–4
Compressive strength (MPa)	10–20 (low strength)	20–40
Bending strength (MPa)	30–50	40–60
Modulus of elasticity in bending (MPa)	3,000–4,000	5,000–7,000
Shear strength (MPa)	2–4 (low)	5–8
End hardness (MPa)	15–25 (soft)	25–35 (harder)
Durability (years)	2–3	5–7
Thermal conductivity (W/m·K)	0.05–0.1 (excellent insulation)	0.1–0.15 (good insulation)

**Table 6:** Comparison of mechanical properties of woods, bamboos, and lianas used in construction of boat-shaped houses

Scientific name	Air-dry Density (g/cm^3^)	Volumetric Shrinkage (%)	Tangential Shrinkage (%)	Radial Shrinkage (%)	Compression Strength (MPa)	Bending Strength (MPa)	Elasticity Modulus (GPa)	Shear Strength (MPa)	End Hardness (MPa)
Wood									
*Litchi chinensis*	0.9–1.0	9–12	5.5–7.0	3.0–4.5	50–60	90–100	9–12	8–12	30–40
*Erythrophleum fordii*	0.9–1.0	12–15	7.0–8.2	4.2–5.0	67–72	140–150	12–13	12–15	60–70
*Tectona grandis*	0.6–0.8	8–12	5.0–6.0	3.0–4.0	60–70	90–120	10–12	12–15	40–50
*Albizia odoratissima*	0.6–0.8	9–12	5.5–7.0	3.5–4.5	40–50	60–80	7–10	5–10	25–35
*Liquidambar formosana*	0.6–0.8	10–12	5.0–6.5	3.0–4.0	50–60	70–90	8–10	8–12	30–40
*Homalium ceylanicum*	0.8–1.0	8–10	5.5–7.0	3.5–5.0	60–80	100–130	11–14	10–15	50–60
*Madhuca hainanensis*	0.7–0.8	10–13	6.0–7.5	4.5–5.5	45–55	90–105	10–11.5	7–10	30–40
*Cocos nucifera*	0.6–0.8	12–15	6.5–8.0	4.0–5.5	40–50	60–75	6.5–8.0	5–10	25–35
*Melia azedarach*	0.6–0.8	10–12	5.0–6.5	3.4–4.5	50–60	70–90	7–9	7–10	30–40
*Bombax ceiba*	0.4–0.6	12–14	7.0–8.5	4.5–5.5	30–40	50–70	6–8	5–8	20–30
*Diospyros strigosa*	0.9–1.1	15–18	8.0–9.5	4.5–6.0	80–95	150–180	16–20	14–18	70–90
Bamboo									
*Bambusa sinospinosa*	0.6–0.7	12–15	6.5–8.0	4.5–5.5	45–55	90–120	7–10	8–12	50–60
*Bambusa textilis*	0.6–0.8	10–14	6.0–7.5	3.5–5.0	40–50	80–110	7.5–9.5	7–11	45–55
*Bambusa pervariabilis*	0.6–0.7	10–13	5.5–7.0	3.0–4.5	35–45	70–100	7–9	6–10	40–50
*Bambusa pallida*	0.5–0.7	9–12	5.0–6.5	3.0–4.0	30–40	60–90	6–8	6–9	35–45
*Bambusa bambos*	0.6–0.8	11–14	6.5–7.5	3.5–5.0	40–55	80–110	8–10	8–12	50–60
*Bambusa xiashanensis*	0.6–0.7	10–13	5.5–7.0	3.0–4.5	35–45	70–100	7–9	6–10	40–50
*Bambusa bambos*	0.6–0.8	11–14	6.5–7.5	3.5–5.0	40–55	80–110	8–10	8–12	50–60
*Bambusa xiashanensis*	0.6–0.7	10–13	5.5–7.0	3.0–4.5	35–45	70–100	7–9	6–10	40–50
Liana									
*Urceola huaitingii*	0.6–0.8	10–15	6.0–9.0	5.0–7.0	45–55	80–100	7.0–8.5	6–8	30–40
*Calamus tetradactylus*	0.7–0.9	12–18	5.0–8.0	4.0–6.0	40–50	60–80	6.5–8.0	5–8	25–35
*Dinochloa multiramora*	0.5–0.7	8–14	7.0–10.0	5.0–7.0	35–50	70–90	6.0–7.5	5–7	20–30
*Dendrocalamus membranaceus*	0.7–0.9	13–17	7.0–8.0	4.5–6.0	50–65	100–130	9–11	10–14	60–70

### Analysis of the thermal environment

#### Indoor and outdoor air temperature trends

Indoor temperature significantly affects human comfort by influencing thermal sensation, productivity, and overall well-being. Field measurements showed clear differences in temperature profiles between traditional boat-shaped houses and modern brick structures [50, 51]. In Chubao Village, during the dry season, the outdoor daytime temperatures ranged from 21 to 27 °C, while the brick houses recorded indoor air temperatures (23.5 °C) approximately 1.3 °C lower than those of the traditional dwellings (24.8 °C). In the rainy season, outdoor temperatures ranged from 27 to 31 °C, with brick houses indoor averaging 0.8 °C hotter than traditional buildings. Among traditional house types, Long Boudoirs exhibited slightly better thermal insulation, being on average 0.9 °C cooler than other traditional boat-shaped houses.

**Fig. 6:**
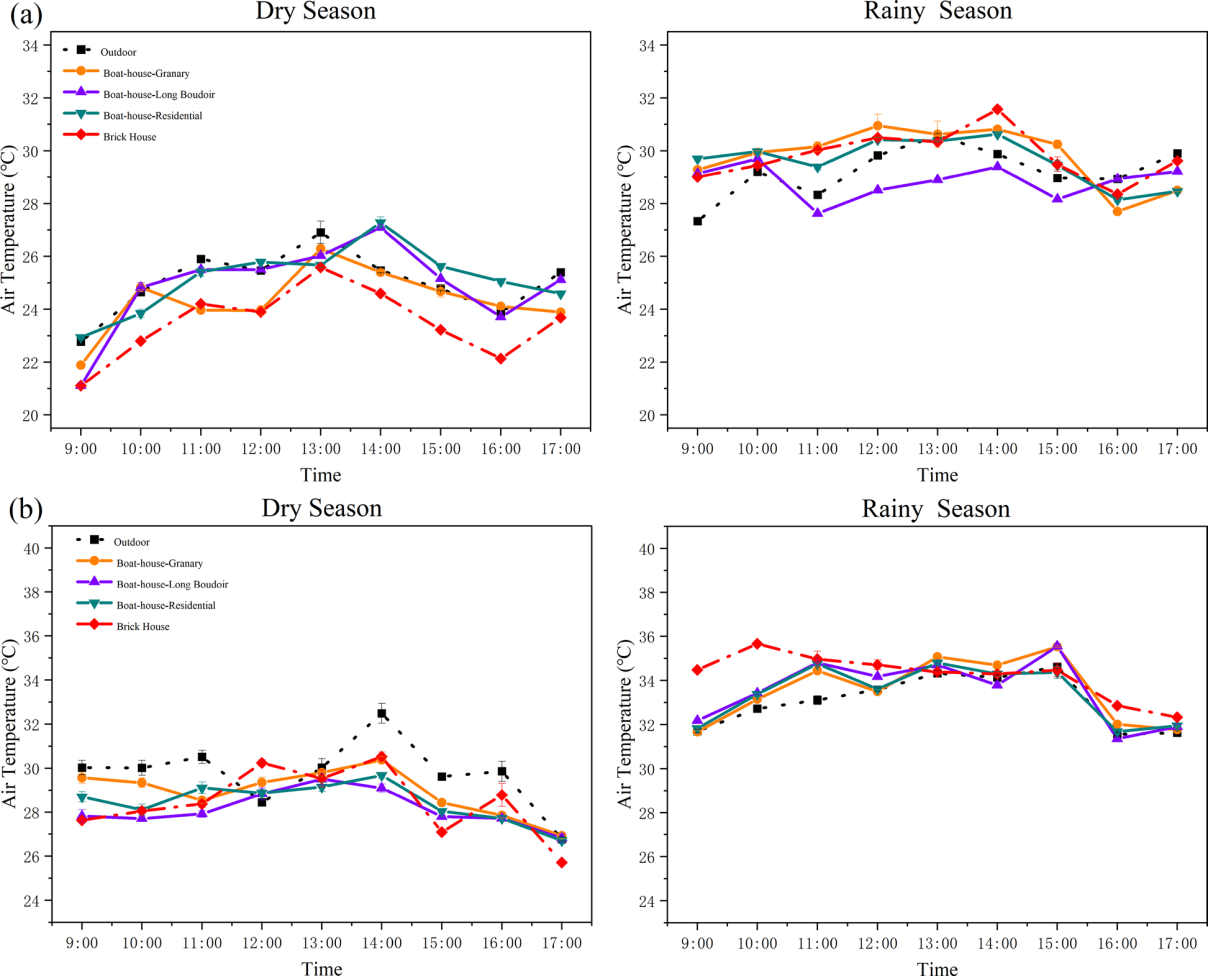
Comparison of air temperature trends of boat-shaped houses between the two traditional Li villages. **a** Chubao Village; **b** Baicha Village

In Baicha Village, daytime temperatures during the dry season ranged from 25 to 33 °C. No significant differences were observed between the indoor temperatures of traditional houses and brick houses. However, in the rainy season (31–35 °C outdoors), the brick houses were consistently 0.8 °C warmer than the traditional houses (Fig. 6).

#### Relative humidity patterns

Relative humidity (RH) was generally higher in the rainy season than in the dry season, as expected. In Chubao Village during the dry season, brick houses had an RH approximately 4.4% higher than traditional boat-shaped houses. The granary recorded RH 4.3% higher than long boudoirs. In the rainy season, the indoor RH of brick houses was 5.5% higher than in traditional buildings, with granaries showing the highest RH among traditional types.

**Fig. 7:**
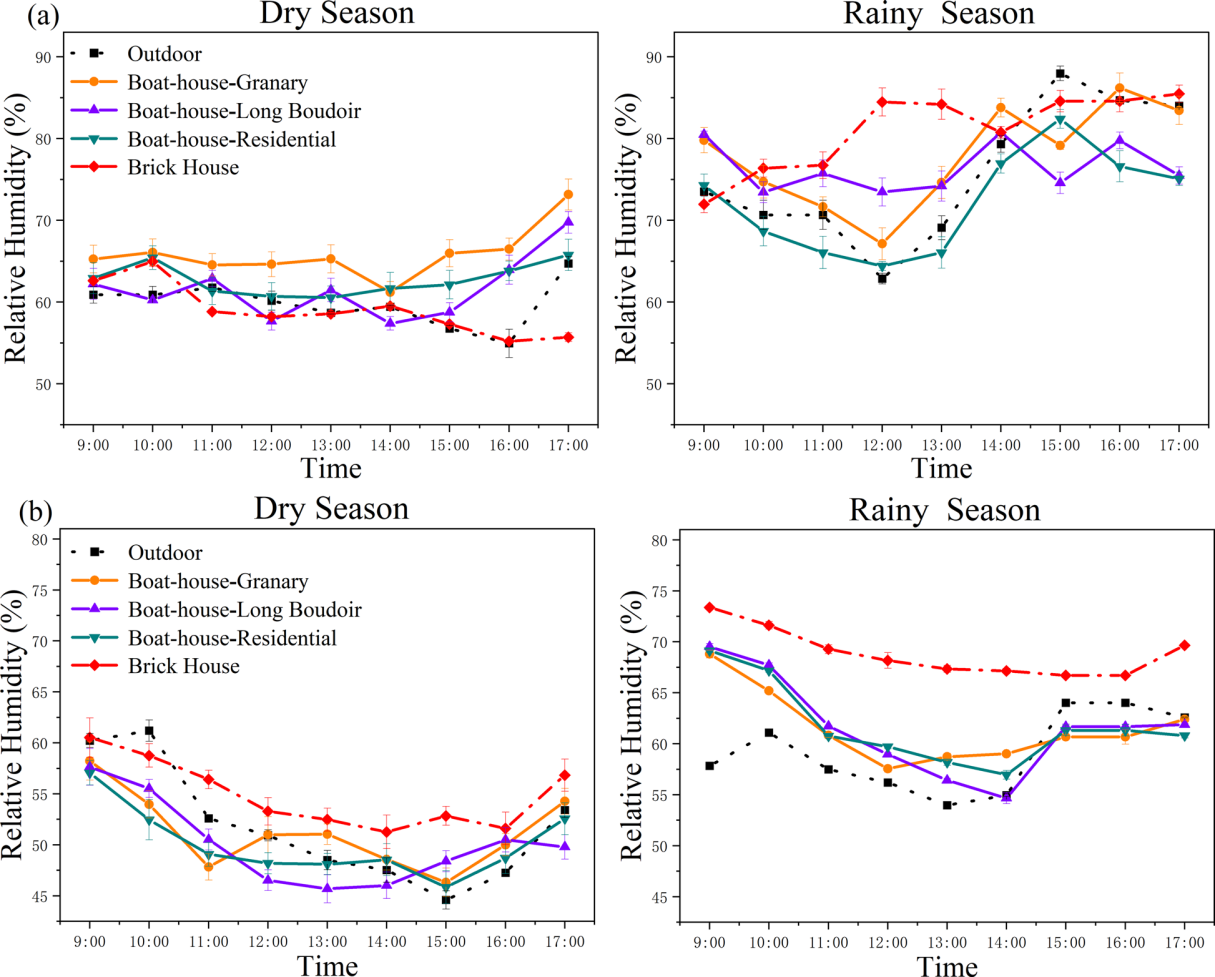
Trends in relative humidity of boat-shaped houses in two traditional villages. **a** Chubao Village; **b** Baicha Village

In Baicha Village, the outdoor dry season RH ranged from 44 to 62%. Interestingly, in this season, brick houses showed slightly lower indoor RH compared to traditional structures (4.4%). However, in the rainy season (outdoor RH 53–64%), indoor RH in brick houses was 7.3% higher than in traditional houses. Differences between traditional house types were minimal (Fig. 7).

#### Comparisons of WBGT (wet bulb globe temperature)

The WBGT values reflect the risk of thermal stress. In Chubao Village during the dry season, all buildings maintained WBGT values below 24 °C, indicating low heat stress for their human occupants. Values for brick houses were 0.8 °C higher than those for traditional buildings. In the rainy season, brick houses recorded WBGT values that averaged 1.1 °C higher than those for traditional structures.

**Fig. 8:**
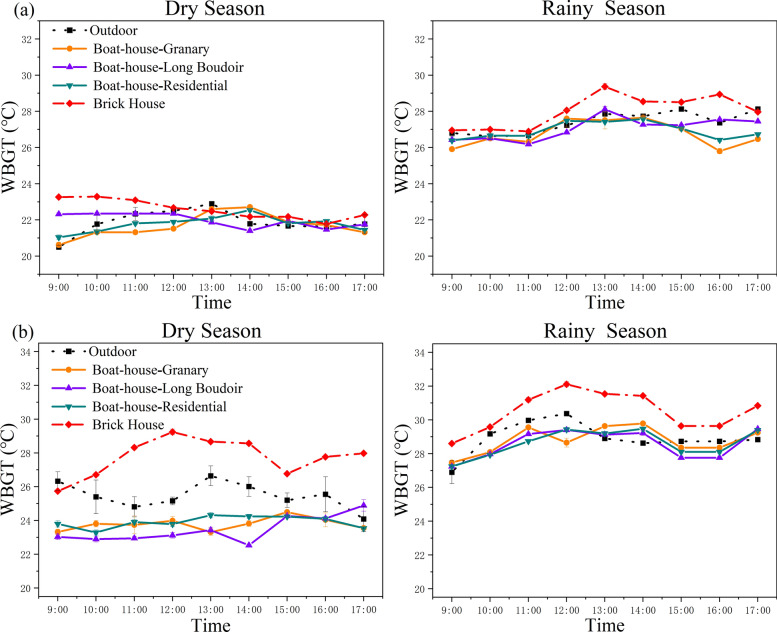
Trends for WBGT values of boat houses in two traditional villages. **a** Chubao Village; **b** Baicha Village

In Baicha Village, the WBGT during the dry season ranged from 24 to 27 °C outside, with brick houses showing WBGT 4 °C higher than traditional buildings. In the rainy season, outdoor WBGT values ranged from 27 to 31 °C, suggesting that human occupants would suffer a moderate to high risk of heat stress. Traditional boat-shaped houses, particularly residential types, performed significantly better than brick houses under natural ventilation, maintaining lower WBGT levels (Fig. 8). These results show that the Li people have cleverly planned and designed the production and living space according to the local natural and geographical conditions in the long-term practice of production and living and made the space as comfortable and livable as possible.

#### Differences in microclimate by altitude in Chubao

**Fig. 9:**
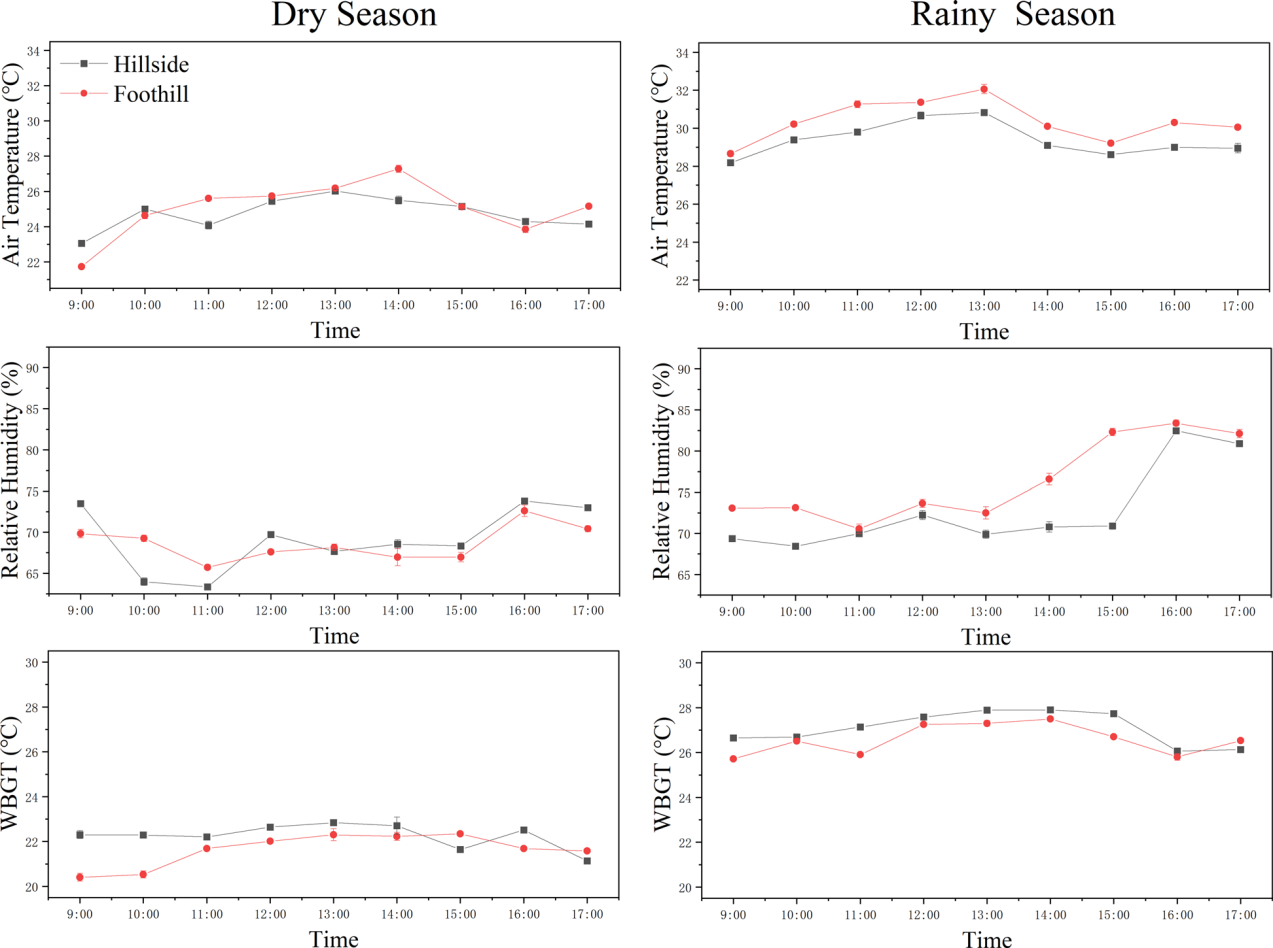
Temperature, humidity, and WBGT values for boat-shaped houses at different altitudes in Chubao Village

In Chubao Village, boat-shaped houses at lower elevations (foothills) showed slightly better thermal performance than those at higher elevations (hillside). During the dry season, the indoor temperatures on the foothill were 0.3° C lower than on the hillside. The WBGT values were 0.6 °C lower in the foothills, while the humidity was slightly higher. However, in the rainy season, foothill houses recorded slightly warmer temperatures (+ 1 °C) and higher humidity (+ 3.6%)(Fig. 9).

#### Differences in microclimates by orientation in Baicha

**Fig. 10:**
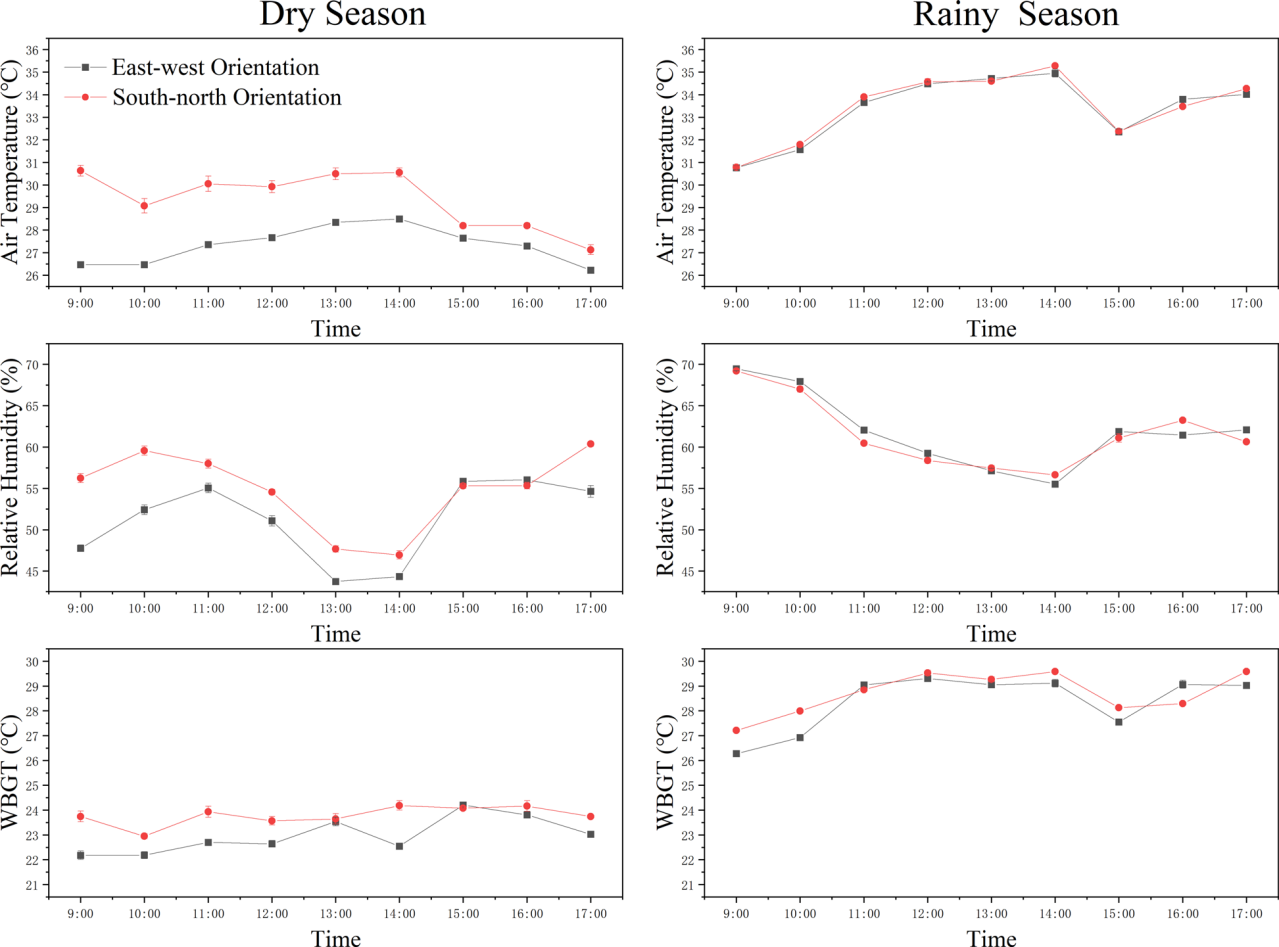
Temperature, humidity, and WBGT trends of boat-shaped houses in different orientations in Baicha Village

In Baicha Village, the orientation of the building influenced indoor environmental conditions. During the dry season, north–south oriented boat-shaped houses had indoor temperatures 2° C higher and WBGT values 0.8° C higher than those oriented east–west. In the rainy season, the differences in temperature and RH were minimal, although WBGT remained slightly higher in the north–south structures (Fig. 10).

#### Independent samples t test analysis of indoor thermal-humidity environment differences among various building types

Variation for measurements in the same type of house, in the same village, and at the same time was relatively small. To ensure clarity and readability of the figures, error bars are not shown. However, standard errors for each measurement are provided in Tables 7 and 8. We compared the daytime indoor temperature, humidity, and wet bulb globe temperature (WBGT) of three types of traditional buildings within two villages. Independent samples t tests were conducted to assess the significance of differences among the three traditional building types, as well as between each traditional type and single-story modern brick houses. Comparisons with non-significant differences were filtered out, and the remaining results are presented in Table 7

**Table 7:** Significance of differences in temperature, relative humidity, and WBGT between two traditional Li villages

Variable	Village	Season	Comparison (A-B)	Mean A	SE A	Mean B	SE B	Mean Diff	SE_combined	t = Diff/SE	Significant
AT(℃)	Chubao	Dry	B-T	23.47	0.45	24.79	0.27	1.31	0.52	2.52	*p* < 0.05
	Chubao	rainy	TL-TGR	28.84	0.22	29.71	0.32	0.87	0.39	2.25	*p* < 0.05
RH(%)	Chubao	Dry	B-T	59.00	1.04	63.39	0.67	4.39	1.24	3.55	*P* < 0.01
	Chubao	Dry	TG-TL	34.24	0.34	33.50	0.26	4.25	0.43	9.88	*P* < 0.01
	Chubao	rainy	B-T	81.02	1.62	75.52	1.10	5.50	1.96	2.81	*P* < 0.01
	Baicha	Dry	B-T	54.89	1.11	50.46	1.22	4.43	1.65	2.68	*P* < 0.01
	Baicha	rainy	B-T	68.89	0.78	61.62	1.32	7.27	1.53	4.74	*P* < 0.01
WBGT(℃)	Chubao	Dry	B-T	22.58	0.18	21.81	0.17	0.77	0.25	3.13	*P* < 0.01
	Chubao	rainy	B-T	28.03	0.30	26.92	0.12	1.12	0.32	3.45	*P* < 0.01
	Baicha	Dry	B-T	27.75	0.38	23.72	0.18	4.03	0.42	9.62	*P* < 0.01
	Baicha	rainy	B-T	30.84	0.42	29.39	0.27	2.05	0.49	4.14	*P* < 0.01

AT—air temperature; RH—relative humidity; B-T means brick house compared to traditional boat-shaped houses (grouping the three types of boat-shaped houses found in each villages; TL-TGR means traditional boat-shaped house of the long Boudoir type compared to traditional boat-shaped houses of both granary and residential types; TG-TL means traditional boat-shaped house of granary type compared to traditional boat-shaped house of the long Boudoir type; and SE—standard error

**Table 8:** Significance of differences in temperature and WBGT according to elevation and orientation

Variable	AT (℃)	WBGT (℃)	AT (℃)	WBGT (℃)
Village	Chubao	Chubao	Baicha	Baicha
Season	rainy	dry	dry	dry
Comparison (A-B)	H-F	H-F	E-N	E-N
Mean A	29.39	22.25	27.33	22.98
SE A	0.30	0.18	0.27	0.24
Mean B	30.36	21.64	29.36	23.78
SE B	0.36	0.24	0.42	0.13
Mean Diff	0.97	0.61	2.03	0.80
SE_combined	0.46	0.30	0.50	0.27
t = Diff/SE	2.09	2.02	4.04	2.94
Significant	*P* < 0.05	*P* < 0.05	*P* < 0.01	*P* < 0.01

AT—air temperature; H-F—hillside boat-shaped house compared to foothill boat-shaped houses (effect of elevation) in Chubao Village; E-N means east–west oriented boat-shaped houses compared to north–south oriented boat-shaped houses (effect of orientation) in Baicha village; SE—standard error

Similarly, we conducted independent samples t test on the differences in building elevation in Chubao Village and building orientation in Baicha Village. After filtering out non-significant comparisons, the results are presented in Table 8.

## Discussion

### Environmental performance of plant-based building materials

The wood species used for beams and columns (e.g., *Erythrophleum fordii*, *Dalbergia hainanensis*, *Madhuca hainanensis*) offer high compressive strength, pest resistance, and dimensional stability. These species are particularly suitable for load-bearing applications in humid conditions, but are now endangered and their use is legally restricted. Bamboo materials (e.g., *Bambusa textilis*, *Dendrocalamus membranaceus*) are valued for their strength–weight ratio, elasticity, low shrinkage, and ease of processing. They are optimal for structural framing and low-carbon applications because of their renewability and availability. Binding materials such as *Calamus tetradactylus* and *C. simplicifolius* exhibit excellent tensile strength and elasticity, essential for flexible and secure joint connections. While *Urceola huaitingii* and *Dinochloa multiramora* are less durable, they remain useful for temporary or interior use. Roof covering materials, such as *Imperata cylindrica* and *Livistona chinensis*, must provide thermal insulation, moisture resistance, ultraviolet durability, and biodegradability. *Livistona chinensis* leaves, due to their waxy surface, outperform *Imperata cylindrica* in water repellency and durability, although both require regular replacement due to weathering and susceptibility to insect attack.

In general, Li boat-shaped house materials demonstrate context-sensitive design: the integration of high-performance materials where needed while balancing affordability, availability, and renewability. Although inferior in absolute mechanical strength compared to modern composites, these natural materials meet the practical needs of architecture in a tropical climate while aligning with principles of low-carbon and biodegradable construction.

### Climate adaptability of boat-shaped house structures

Comparative thermal analysis reveals that traditional boat-shaped houses outperform modern brick houses in regulating indoor environments under natural tropical conditions.

The thatched roofs, porous walls, and cross-ventilation designs inherent in boat-shaped houses provide superior passive cooling and moisture regulation. Double-gable entrances and elevated roof structures improve airflow and mitigate thermal accumulation. Natural plant materials with hygroscopic properties also contribute to humidity control and thermal buffering.

Among the different forms of boat-shaped houses, the plant-and-mud wall houses in Baicha demonstrated better insulation and ventilation compared to the plant-only houses in Chubao. The long boudoir exhibited the lowest humidity levels in the rainy season, probably due to its smaller size and better airflow. The granary, mainly used for rice storage, prioritized ventilation and heat radiation, minimizing mold and rot.

These findings indicate that spatial organization and material selection in boat-shaped house construction were guided by sophisticated environmental knowledge, allowing the structures to remain cool and dry throughout the seasons (Table 7).

### Influence of elevation and orientation on the thermal environment

Microtopography and orientation significantly influence the thermal environment of traditional houses. In Chubao, houses at lower elevations remained cooler and more humid, benefiting from valley airflow and the proximity of groundwater. On the contrary, the hillside structures were drier but slightly warmer due to solar exposure and reduced canopy cover.

In Baicha, houses oriented north–south experienced higher temperatures and WBGT values, probably due to prolonged solar exposure. East–west orientation offered better shading and airflow under local wind regimes, confirming previous research on optimal building orientations in tropical climates.

### Drainage and humidity management strategies

Effective drainage and dehumidification systems are essential for structural durability and health of human occupants in the humid tropics. The boat-shaped houses in Chubao used sloped floors and stone-lined channels that directed water into a village-wide canal system. Baicha’s relatively flat topography required surface trenches leading to rice paddies or distant rivers.

Thatched roofs with extended eaves, layered leaf design, and elevated platforms facilitated rapid rainwater runoff and drying. The use of breathable materials such as bamboo and thatch allowed natural dehumidification, reducing the risk of fungal growth.

Mud walls with embedded straw further buffered humidity while moderating internal temperature fluctuations, demonstrating an integrated water management approach embedded in local architectural traditions.

### Social and cultural influence of boat-shaped houses

The boat-shaped houses exemplify an old integration of tropical building structural design, spatial logic, and cultural expression. Architecturally, their elongated, arched-roof form mimics an inverted boat, providing resilience against tropical rains and symbolizing the seafaring origins of the Li people. The internal spatial layout is not only practical but also deeply symbolic—zones are organized according to family hierarchy, gender roles, and ritual functions, reinforcing social order and kinship ties within the household.

Daily life within these structures reflects a communal lifestyle, where multiple generations coexist under one roof, sharing resources and responsibilities. The use of locally sourced materials such as bamboo, rattan, and thatch demonstrates ecological wisdom, enabling natural ventilation, temperature regulation, and harmony with the surrounding tropical rainforest. Furthermore, the construction of different dwelling types relies on traditional craftsmanship passed down through generations, particularly in traditional techniques, preserving both material knowledge and intangible cultural heritage.

Together, these aspects position the Li boat-shaped house as more than a dwelling—it is a living manifestation of Li cosmology, environmental adaptation, and intergenerational continuity, offering rich insights for studies in vernacular architecture, anthropology, and sustainable design. These nuanced differences emphasize the importance of incorporating culturally specific plant uses into conservation and sustainable development frameworks. Integrating locally valued species such as *Calamus* and *Madhuca* into ecotourism and sustainable handicraft initiatives can reinforce community engagement and preserve intangible cultural heritage. Moreover, recognizing the symbolic and ritual significance of certain plants enhances the development of culturally sensitive conservation strategies that resonate with indigenous worldviews. This integrative approach supports both biodiversity conservation and the maintenance of traditional knowledge systems amid rapid environmental and social change.

However, in our interviews, many elders expressed with a sense of regret that, with rapid economic development, the boat-shaped houses—once built to achieve thermal comfort through natural means—have gradually been phased out, replaced by rows of uniform brick houses in the newly developed rural settlements. At the same time, mainstream cultures from mainland China and the West have spread widely through the internet, leading many Li people to yearn for the excitement and prosperity of urban life. As a result, their emotional attachment to the boat-shaped houses and their own traditional culture has gradually faded.

Although our measurements demonstrate the superior daytime thermal performance of traditional boat-shaped houses, the lack of night-time data limits our ability to assess passive cooling performance during the full diurnal cycle. Previous studies have highlighted the importance of night ventilation in tropical climates, and future studies should include night-time monitoring to better understand thermal comfort under such conditions [48–51].

## Conclusions

The boat-shaped house of the indigenous Li people is a unique architectural form that has evolved over thousands of years in response to the tropical rainforest environment of Hainan Island. Shaped by ecological and cultural factors, this traditional type of building exemplifies sustainable design, community resilience, and the deep integration of human and natural systems.

Using locally available plant materials, many of which possess superior mechanical and ecological properties, the Li people have developed a construction system that is lightweight, low cost, low carbon, and highly adapted to the regional climate. Our findings demonstrate that traditional boat-shaped houses offer better thermal comfort, humidity control, and mitigation of heat stress under natural conditions than do modern brick houses. These benefits are especially pronounced during the hot and humid rainy season.

Beyond physical performance, boat-shaped houses also reflect an intangible cultural heritage, embodying beliefs about nature, community organization, gender roles, and environmental stewardship. However, modernization and urban migration are accelerating the loss of traditional knowledge and the abandonment of these structures.

To ensure the survival of this heritage, urgent action is needed to document, protect, and adapt the boat-shaped house system within contemporary development strategies. The architectural principles underlying the boat-shaped house—passive design, material renewability, environmental fit—can inform modern ecological architecture in tropical regions. Integrating traditional wisdom with modern technologies offers a pathway to resilient, low-carbon, and culturally meaningful housing.

Finally, this study supports efforts of local and national stakeholders to nominate the “Boat House and Traditional Settlements of the Li ethnic minority” as a UNESCO World Natural and Cultural Heritage Site. By demonstrating the ecological, architectural, and cultural value of these settlements, our work contributes to the broader goal of preserving both biodiversity and cultural diversity in tropical rainforest landscapes.

## Supplementary Information

Additional file 1.

## Data Availability

No datasets were generated or analysed during the current study.

## Abbreviations

TK: Traditional knowledge
WBGT: Wet bulb globe temperatures
IUCN: International Union for Conservation of Nature
UNESCO: United Nations Educational, Scientific and Cultural Organization

## Declarations

### Content for publication

No applicable.
